# Supplementary material for: Joint duration-cost-quality optimization model for complex product supply chains under contingency conditions
Source: PLoS One. 2023 Oct 16;18(10):e0292010. doi: 10.1371/journal.pone.0292010 (PMC10578608; doi:10.1371/journal.pone.0292010)
Supplement: Attachment — Submitted filename: Response to Reviewers.docx [file pone.0292010.s001.docx]

Dear Editor Ibrahim Badi and reviewers,

Thank you for your letter and the reviewers’ comments on our manuscript entitled “Joint duration-cost-quality optimization model for complex product supply chains under contingency conditions” (ID: PONE-D-23-15329). Those comments are very helpful for revising and improving our paper, as well as the important guiding significance to other research. We have studied the comments carefully and made corrections which we hope meet with approval. The main corrections are in the manuscript and the responds to the reviewers’ comments are as follows(the replies are highlighted in blue).

Replies to the reviewers’ comments:

Reviewer #1:

Comment 1: It might be best if you numbered 3 objectives (1. The first is to meet the requirements of shortening the duration, saving cost and guaranteeing quality of complex products under emergency situations, so as to respond to the delivery demand quickly and efficiently. 2. The second is to improve and enrich the theoretical research related to the supply chain of complex products, especially the content related to the supply chain of complex products in emergency situations. 3. The third is to provide decision basis for decision makers in the actual manufacturing of complex products based on the model findings.)

Response: We think your suggestions are sound and will make the content more accessible and better organised. We have numbered the objectives as you suggested.

Comment 2: Error - The period at the end of the sentence is missing (mergency situationsThe third is to provide).

Response: Thank you for pointing out this detail for us, which was due to our negligence. We will have to pay more attention to these details in our future academic research. Once again, we would like to express our gratitude to you. We have corrected this error.

Comment 3: What are the currencies of the costs? Please indicate the currency in the entire text. (450 thousands to 640 thousands).

Response: We are using RMB as the unit of currency. Thank you for your patience and care in helping us point out the shortcomings in the article. We have now clarified the currency units in the article.

Comment 4: My main comment and basic recommendation for improving the paper is to write the basic information in chapter five and to expand the basic input information about the case study. These data should be described in detail in terms of what they mean and what they are about. In the way it is now presented, I could not fully understand all the information.

Response: Thank you for pointing out this deficiency for us. We have followed your suggestion and added a presentation of basic information to the case study, including the production processes and mechanisms of complex products in the supply chain.

Reviewer #2:

Comment 1: Please make each sentence within the abstract very short and comprehensive instead of lengthy sentence.

Response: Thank you for your patience in teaching us these practical essay writing tips. We have followed your advice and turned the lengthy sentences in the abstract into short ones.

Comment 2: Please, clearly state the motivations and contributions of your study.

Response: (1) Our motivations: A complex supply chain network is gradually formed through collaboration and innovation between main manufacturers and suppliers, with products as the link. A problem with any one supplier in the network can have a significant impact on the entire complex product supply chain, affecting delivery times, product quality and costs of complex products. Therefore, in order to ensure that complex products are delivered quickly, at a lower cost, and with high quality in emergency situations, we have studied the supply chain of complex products in emergency situations.

(2) Contributions: Researches on complex product-related issues have produced some significant results, but it has also revealed the following problems: (a) In reality, a main manufacturer often works with multiple suppliers to form a network of supply chains, and many studies of complex products based on game theory from a contractual perspective are limited to one main manufacturer and one supplier, which has certain limitations. (b) When studying problems related to complex products based on GERT network models, most models have deterministic values for network parameters such as product quality, delivery time and product cost, which do not correspond to the actual situation where such parameters are non-deterministic. In some cases, although the non-deterministic nature of the network parameters is taken into account, the model is limited to the individual enterprise and is not applied to the supply chain network. (c) there is currently little exploration of complex product supply chains in emergency situations. In conclusion, the existing literature only explores revenue sharing, quality incentives, pricing strategies or elements related to a specific stage of the supply chain for complex products. Taking these into account, this paper presents a joint duration-cost-quality optimisation study of complex product supply chains in contingency situations and is therefore a further study that complements and enriches existing research.

Comment 3: Please, make an extensive review of past studies related to complex product supply chains networks in terms methodology used, location, objective and compare them to your current study. A provision of summarized Table to highlight these studies will be very good presentation. Besides, it is requested that recent studies to be used as the ones used (references 17-20, 22, 23) within your study are very old and some dated back to 1987. References less than 5 years of publication are suggested (2018 to now).

Response: We have re-studied and researched the relevant literature and have now updated our citations to the last 5 years of literature, and have provided a summarized Table to highlight these studies as suggested.

Comment 4: Please what is the advantage of the methodology applied in your study and why did you choose it rather than other existing methods.

Response: Project management tools, represented by critical path (CPM), Program Evalution and Review Technique (PERT), consider probabilistic uncertainty and are better suited to a wide range of areas such as analytical modelling, development management, quality management, reliability assessment and resource allocation. However, for the many non-probabilistic uncertainty problems that exist in reality, these management tools are no longer applicable. Compared with the CPM, PERT, the GERT network model has wider application fields, such as space research, cost optimization management, emergency plan management, project risk management, product development, manufacture and improvement plan, combat optimization problem, and computer algorithms for GERT networks. The GERT network model has become a powerful tool for dealing classical probabilistic network relations.

Comment 5: I suggested authors to numbering the equations within the manuscript as 1, 2, 3,.., etc.

Response: Thank you for your suggestion, we have numbered the equations as suggested.

Comment 6: Please, uniform the size of writing style for Figures titles as well as Tables.

Response: Thank you for pointing out this detail for us, which was due to our negligence. We will have to pay more attention to these details in our future academic research. Once again, we would like to express our gratitude to you. We have uniformed the size of writing style for Figures titles as well as Tables.

Comment 7: The conclusion is too lengthy. I suggested authors to just highlight the main findings, the limitations of the study as well those of the method used. Also, authors should give some recommendations for future studies.

Response: Thank you for your suggestion, we have simplified the conclusion as you suggested and given some recommendations for future studies. In the future, we will also learn from this experience and just highlight the main findings, the limitations of the study as well those of the method used.

Comment 8: The linguistic quality needs to be polished and several grammar mistakes need to be revised in the full context.

Response: In response to the question, we carefully read through the article and meticulously polished the language in the article. Again, thank you again for your meticulous and patient advice and teaching skills. We will take this opportunity to reflect and summarise our shortcomings seriously, and strive to refine our research and make more achievements, so as to make our due contribution to the progress and development of all mankind and society.

Once again, thank you very much for your constructive comments and suggestions which would help us both in English and in depth to improve the quality of the paper.

Kind regards,

Peng Dong

E-mail: [0913041005@nue.edu.cn](mailto:0913041005@nue.edu.cn)

Corresponding author: Peng Dong

E-mail address: [0913041005@nue.edu.cn](mailto:0913041005@nue.edu.cn)
