# Supplementary material for: Joint duration-cost-quality optimization model for complex product supply chains under contingency conditions
Source: PLoS One. 2023 Oct 16;18(10):e0292010. doi: 10.1371/journal.pone.0292010 (PMC10578608; doi:10.1371/journal.pone.0292010)
Supplement: Attachment — Submitted filename: Response to Reviewers.docx [file pone.0292010.s002.docx]

Dear Academic Editor and reviewers,

Thank you again for your letter and the reviewers’ comments on our manuscript entitled “Joint duration-cost-quality optimization model for complex product supply chains under contingency conditions” (ID: PONE-D-23-15329). Those comments are very helpful for revising and improving our paper, as well as the important guiding significance to other research. We have studied the comments carefully and made corrections which we hope meet with approval. The main corrections are in the manuscript and the responds to the reviewers’ comments are as follows(the replies are highlighted in blue).

Replies to the Journal Requirements:

Journal Requirements: Please review your reference list to ensure that it is complete and correct. If you have cited papers that have been retracted, please include the rationale for doing so in the manuscript text, or remove these references and replace them with relevant current references. Any changes to the reference list should be mentioned in the rebuttal letter that accompanies your revised manuscript. If you need to cite a retracted article, indicate the article’s retracted status in the References list and also include a citation and full reference for the retraction notice.

Response: Thank you very much for your suggestion, we have checked the cited literature and corrected the problematic areas, which are marked in the list of literature in the text.

Replies to the reviewers’ comments:

Reviewer #1:

Comment 1: A reference number cannot be the beginning of a sentence in this way. Write the last name of at least the first author.

Response: Thank you very much for your patience and sincerity in pointing out the errors for us. We have already corrected the errors you pointed out and re-checked all the relevant positions again and also made corrections and additions to the three places [10], [17], [18].

Comment 2: Explain abbreviations when they are mentioned for the first time in the text. (“BA small-world networks or ER”).

Response: Thanks again for your patience and help, we have explained the meaning of the abbreviations by explaining them in the text.

Comment 3: These sentences seem unfinished to me. Please elaborate on these sentences.(Page 35–“ Where node 1 is a normalised virtual source node representing the layer 2 supplier. Nodes 2, 3 and 4 represent three layer 1 suppliers. Node 5 represents a main manufacturer, and node 6 represent a customer. In the supply chain network, node 1”).

Response: We are very sorry for any inconvenience caused while you were reviewing the article, and we have reintroduced additional clarification in the text of what the nodes represent in the network model.

Reviewer #2:

The authors have addressed all the comments. The manuscript is technically sound and presented in an intelligible fashion

Response: Thank you for your suggestions on our articles and for recognising our efforts. We value this opportunity to learn and we will continue to work hard.

Once again, thank you very much for your constructive comments and suggestions. We treasure and value this opportunity. Upon receipt of the review comments, we immediately made revisions and improvements in the hope that this time we can meet the requirements and approval.

Kind regards,

Peng Dong

E-mail: [0913041005@nue.edu.cn](mailto:0913041005@nue.edu.cn)

Corresponding author: Peng Dong

E-mail address: [0913041005@nue.edu.cn](mailto:0913041005@nue.edu.cn)
